# Interleukin-22 Influences the Th1/Th17 Axis

**DOI:** 10.3389/fimmu.2021.618110

**Published:** 2021-02-22

**Authors:** Hannes Lindahl, Tomas Olsson

**Affiliations:** Department of Clinical Neuroscience, Karolinska Institutet, Stockholm, Sweden

**Keywords:** interleukin 22 (IL-22), interferon gamma, interleukin 17 (IL-17), animal models, inflammatory disease, infectious disease

## Abstract

Interleukin-22 (IL-22) is secreted by a wide range of immune cells and its downstream effects are mediated by the IL-22 receptor, which is present on non-immune cells in many organs throughout the body. IL-22 is an inflammatory mediator that conditions the tissue compartment by upregulating innate immune responses and is also a homeostatic factor that promotes tissue integrity and regeneration. Interestingly, the IL-22 system has also been linked to many T cell driven inflammatory diseases. Despite this, the downstream effects of IL-22 on the adaptive immune system has received little attention. We have reviewed the literature for experimental data that suggest IL-22 mediated effects on T cells, either transduced directly or *via* mediators expressed by innate immune cells or non-immune cells in response to IL-22. Collectively, the reviewed data indicate that IL-22 has a hitherto unappreciated influence on T helper cell polarization, or the secretion of signature cytokines, that is context dependent but in many cases results in a reduction of the Th1 type response and to some extent promotion of regulatory T cells. Further studies are needed that specifically address these aspects of IL-22 signaling, which can benefit the understanding and treatment of a wide range of diseases.

## Introduction

Interleukin-22 (IL-22) is often described as a cytokine that is expressed by immune cells but that exclusively acts on non-immune cells (1, 2). Its role is best understood at so called barrier surfaces such as the skin, lungs, and gut where the effects of IL-22 ligation typically involve proliferation, regeneration, or activation of innate immune mechanisms. Interestingly, the IL-22 system has also been linked to a range of T cell driven inflammatory diseases such as rheumatoid arthritis (RA), graft *versus* host disease (GvHD), and multiple sclerosis (MS) (2–4). However, relatively little is known about how IL-22 influences T cell polarization. The fact that the IL-22 receptor (IL-22R) has mostly been shown to be absent on immune cells has likely contributed to this (1). Although there are an increasing number of reports of IL-22R expression on immune cells, in most cases any effect of IL-22 on T cells is likely indirect, transduced by mediators originating from IL-22 receptor expressing non-immune cells. IL-22 biology in general has been comprehensively described in excellent review articles (2, 4). Here, we have reviewed the literature for data that suggest effects of IL-22 on T helper cell polarization or secretion of the signature cytokines IFNγ and IL-17. Any experimental setup where IL-22 signaling has been specifically targeted has been screened for such data. By applying this perspective to the literature, we hope to promote further research on the role of IL-22 in shaping adaptive immunity in inflammatory and infectious diseases.

## Interleukin 22

IL-22 was initially described as a cytokine produced by IL-9 activated T cells (5) and was later associated with the Th17 lineage (6). It has now become clear that IL-22 is not only frequently produced independently of IL-17 (7–9) but can also be produced by a wide range of other immune cells (**Table 1**). There are also reports of IL-22 being produced by non-hematopoietic cells (35, 36).

**Table 1:** Cell types that express IL-22.

Cell type	Comments
Th1	Th1 polarization of human peripheral blood T cells *in vitro* induces IL-22 production (10).
Th17	In mice, expression of IL-22 is higher in Th17 cells compared to Th1 and Th2 (6, 11, 12). Colonization of germ-free mice with segmented filamentous bacteria promotes Th17 cells in the lamina propria of the small intestine (13).
Th22	Th22 is a distinct T helper subset that does not produce IFNγ, IL-4 or IL-17. It expresses CCR6 and skin homing CCR4 and CCR10. Differentiation is promoted by IL-6, TNF, and ligation of the AhR (7–9). *In vitro* and *in vivo*, Th22 cells show a marked plasticity toward IFNγ production under Th1 polarizing conditions (14).
CD8 T cells	IL-17 producing cytotoxic T cells often co-produce IL-22 (15–17).
γδ T cells	γδ T cells are an early source of IL-22 in several disease models (18, 19).
MAIT cells	MAIT cells from the female genital tract preferentially produce IL-17 and IL-22 after microbial stimulation in contrast to blood MAIT cells, which primarily produce IFNγ, TNF, and granzyme B (20).
NKT cells	Murine splenic NKT cells produce IL-22 after activation *in vitro* (21). IL-22 and IL-17 production by human NKT cells is largely segregated, implying distinct roles (22).
ILC3	NKp46^+^ RORγt^+^ ILCs are present in the intestines and are a source of IL-22, which has important local homeostatic and protective effects (23–26).
LTi cells	LTi cells are a rapid innate source of IL-22 involved in the development of secondary lymphoid tissue (27, 28).
Macrophages	IL-22 producing macrophages have been described in psoriatic skin (29).
Dendritic cells	*Ex vivo* and bone-marrow derived dendritic cells can express IL-22 in response to IL-23 (30, 31).
Neutrophils	Neutrophils can produce IL-17 and IL-22 in response to IL-23 (32, 33).
Mast cells	A subset of mast cells isolated from human skin affected with psoriasis or atopic dermatitis produce IL-22 (34).
Non-hematopoietic cells	Acinar cells of the lacrimal glands are the primary source of IL-22 in a mouse model of dry eye disease (35).

TNF, tumor necrosis factor; AhR, aryl hydrocarbon receptor; IFNγ, interferon gamma; γδ T, gamma delta; MAIT cells, mucosa associated invariant T cells; NKT cells, natural killer T cells; RORγ t, RAR-related orphan receptor gamma t; ILC, innate lymphoid cells; LTi cells, lymphoid tissue inducer cells.

The most well-established inducer of IL-22 is IL-23 (6, 30). The IL-23 receptor has been detected on Th17 cells, natural killer T (NKT) cells, type 3 innate lymphoid cells (ILC3), gamma delta (γδ) T cells, macrophages, dendritic cells (DC), and neutrophils. Stimulation of these cells with IL-23 can induce production of IL-22 (11, 18, 29, 31, 32, 37, 38). Moreover, IL-1β can act both independently of and synergistically with IL-23 to induce IL-22 (18, 37, 39). IL-6 and TNF polarize naïve T cells to the Th22 lineage (8). The aryl hydrocarbon receptor (AhR) promotes the differentiation of several IL-22 producing cells such as Th17 cells, Th22 cells, and ILC3 (40, 41). Ahr is located in the cytoplasm where it senses endogenous and exogenous ligands, leading to nuclear translocation and transcription of *IL22* and other genes (41–43). Similarly, transcription factor RAR-related orphan receptor gamma t (RORγt) is also necessary for the differentiation of Th17 cells and ILC3 (40, 44). Furthermore, IL-22 induction by Toll-like receptor (TLR)2 ligation has also been described in innate lymphoid cells (45, 46).

**Figure 1:**
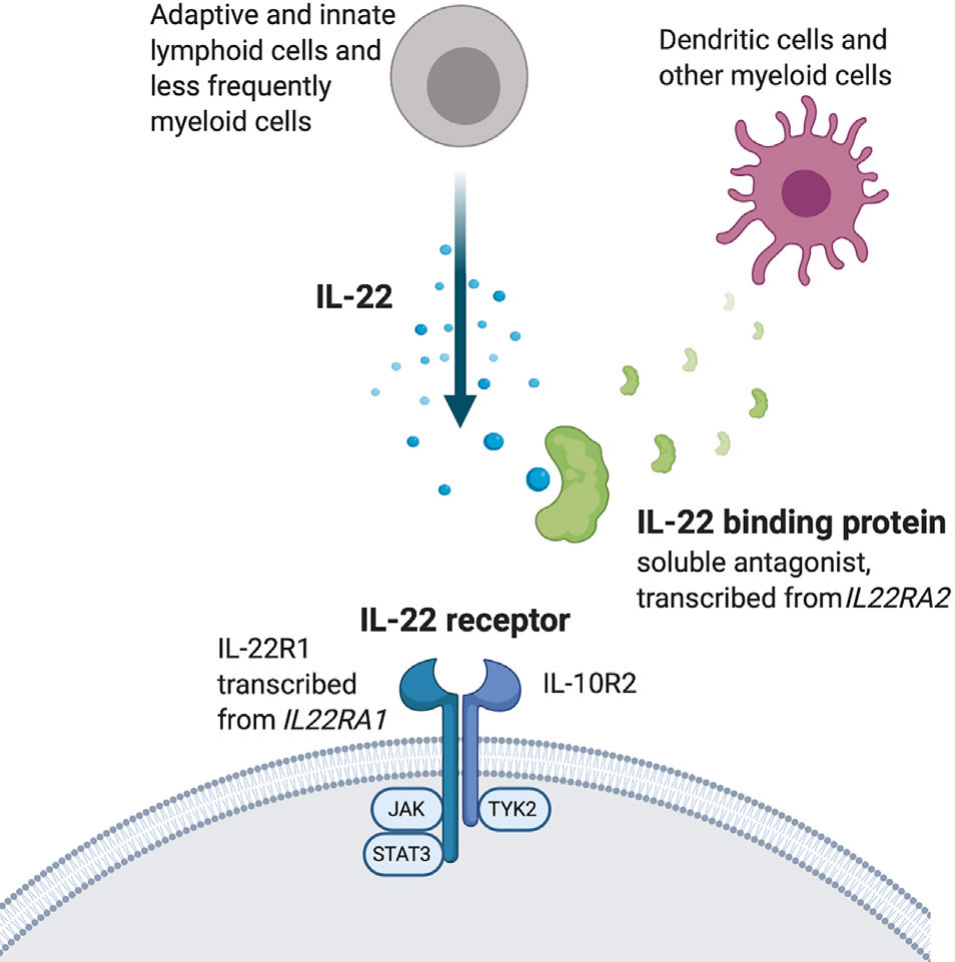
The IL-22 system.

Negative regulators of IL-22 production include transforming growth factor beta (TGF-β), IL-27, and Inducible T cell co-stimulator (ICOS), all of which transduce signals to the transcription factor c-Maf (47–49). Interestingly, IL-22 is one of few cytokines that has a dedicated soluble antagonist molecule to regulate its effects *in vivo*, IL-22 binding protein (IL-22BP), transcribed from the gene *IL22RA2* (**Figure 1**) (50, 51). Insights about the role of IL-22 can therefore also be gained from experiments in which IL-22BP levels have been manipulated.

### Interleukin 22 Receptor

The IL-22R is a heterodimer of the subunit IL-10R2, which is expressed by most cells, and the more selectively distributed subunit IL-22R1 (**Figure 1**) (52–56). Ligation of IL-22R with IL-22 induces activation of the tyrosine kinases JAK1 and Tyk2, which in turn activates STAT3. STAT1 and STAT5 activation has also been reported (1, 57) as well as downstream signaling *via* the MAPK pathways (36, 58–60). IL-22R is expressed by epithelial and parenchymal cells in a wide range of tissues throughout the body. It is highly expressed at, but not limited to, barrier surfaces such as the skin, gut and lungs. Other tissues where IL-22 exerts effects include liver, thymus, pancreas, kidney, and synovium (25, 36, 61–63).

IL-22R has mostly not been detected on immune cells, which has established IL-22 as a cytokine that mediates one-way signaling from immune cells to tissue cells. However, there are reports of IL-22R expression on both myeloid and lymphoid cells and functional data on the effects of IL-22 on these cells. IL-22 has direct effects on splenic CD4 T cells, B cells and CD11b^+^ cells in a mouse model of autoimmune arthritis (64–67) and on splenic CD11b^+^ cells in a mouse model of autoimmune uveitis (68). Moreover, infiltrating mononuclear cells in the salivary glands of patients with primary Sjogren’s syndrome express IL-22R1 shown by immunohistochemistry (69). Further characterization by flow cytometry showed IL-22R expression on macrophages and, to a lesser extent, on T and B cells both in salivary glands and in the circulation from primary Sjogren’s syndrome patients but IL-22R was not detected at all in samples from non-specific chronic sialoadenitis. During the acute stage of *Mycobacterium tuberculosis* infection in mice, IL-22R is expressed primarily on epithelial cells. However, during the chronic stage both epithelial cells and recruited macrophages express IL-22R, which also was observed in human samples (46). IL-22 inhibits intracellular growth of *M tuberculosis* in human monocyte derived macrophages (70). Stimulating peripheral blood mononuclear cells from primary Sjogren’s syndrome patients *in vitro* with IL-22 increased production of IL-17, which was not seen in non-specific chronic sialoadenitis (69). CD14^+^ adipose tissue macrophages, but not circulating CD14^+^ cells, express IL-22R1 shown by western blot and FACS (71). IL-22 strongly induces IL-1β from CD14^+^ adipose tissue macrophages.

## The Influence of Interleukin-22 on the Th1/Th17 Axis

### Inflammatory Diseases

#### Uveitis

The animal model experimental autoimmune uveitis (EAU) can be induced by immunization with retinal autoantigens and disease severity is evaluated by fundoscopy. Treatment with IL-22 before onset of EAU in mice results in reduced severity of disease and delayed onset (68). The protective effect of IL-22 in this model is associated with an overall reduction in eye-infiltrating cells with a proportional decrease in T cells and neutrophils. Interestingly, the IL-22R subunit *Il22ra1* is highly expressed by splenic CD11b^+^ cells day 12 after immunization. When stimulated *in vitro* with IL-22, these cells produce less IL-6, IL-12, IL-23, and IL-1β but more IL-10 and TGF-β. Consistent with a tolerogenic phenotype, they also express less MHC class II, CD80, CD86, and CD40 but more PD-L1. IL-22 treated APCs yield less antigen specific T cell proliferation *in vitro* and induce less IFNγ and IL-17 production but more IL-10 production.

#### Dry Eye Disease

Dry eye disease is predominantly a Th17 cell driven autoimmune disorder resulting in ocular mucosal inflammation, which in severe cases can lead to damage to the cornea and vision loss. Both IL-17 and IL-22 are elevated in tear and lacrimal fluid in persons with dry eye disease (35). IL-17 levels are positively correlated and IL-22 levels negatively correlated with severity of disease. In a mouse model of dry eye disease IL-22 neutralizing antibody or *Il22* gene deletion both result in increased infiltration of Th17 cells and more severe disease, consistent with a protective effect of IL-22. IL-22R is expressed on the ocular surface and *in vitro* stimulation of corneal epithelial cells with IL-22 inhibits expression of inflammatory mediators, including the Th17-inducing cytokines IL-6 and IL-23.

#### Colitis

The homeostatic role of IL-22 in the gastrointestinal tract has received much attention (72). Activation of AhR alone promotes IL-22 secretion by intestinal leukocytes but it also acts synergistically with transcription factor RORγt (40). Steady state *Ahr* ^−/−^ mice have an increased number of intestinal Th17 cells as a result of commensal segmented filamentous bacteria expanding and locally inducing Th17 cells (73). Administration of IL-22 to *Ahr* ^−/−^ reduces Th17 cells to numbers that approach normal levels. The increased intestinal Th17 numbers in *Ahr* ^−/−^ mice do not lead to any overt gut pathology. However, when one allele of the RORγt gene is deleted in *Ahr* ^−/−^ mice IL-22 production is further decreased, which is associated with occasional observations of spontaneous colitis. The authors of this study suggest that in immunocompromised patients, that potentially have impaired IL-22 production, the normally innocuous segmented filamentous bacteria may expand and cause intestinal autoimmunity through induction of pathogenic Th17 cells.

#### Arthritis

Collagen induced arthritis (CIA) is an animal model of RA. Daily administration of recombinant IL-22 starting before onset of arthritis reduces disease severity of CIA in DBA mice but does not alter incidence (74). The IL-22 treatment increases expression of IL-10 in the spleen and administration of neutralizing IL-10 antibody together with IL-22 cancels the protective effect of the latter. IL-22 induces secretion of IL-10 from CD11b^+^ splenocytes harvested during early disease. Addition of IL-22 decreases IFNγ secretion induced *in vitro* by polyclonal T cell activation of splenocytes harvested from mice with early disease as well as from naïve mice. In contrast, stimulation with IL-22 increases IL-17 secretion from splenocytes restimulated *in vitro* with collagen.

In a follow-up study, Justa et al. showed that IL-22 has a dual role in CIA. Treatment with a neutralizing IL-22 antibody before onset of arthritis increases disease severity consistent with the previous study, whereas treatment after onset reduces disease severity (64). Surprisingly, they show that after disease onset IL-22 actually decreases *in vitro* IFNγ production from splenocytes after restimulation with collagen. No effect on IL-17 or IL-10 was observed in this context. However, IL-22 causes increased proportions of Th1 cells in the draining lymph nodes but still does not influence Th17 cells. In the affected joints the proportions of Th17 cells are increased as result of IL-22 signaling. *Il22ra1* mRNA and IL-22R1 protein expression is detected in CD4^+^ splenocytes from mice with arthritis but not at baseline or during the initiation phase. CD11c^+^ cells and CD4 T cells from the spleen of arthritic mice co-cultured in the presence of recombinant IL-22 results in less production of IFNγ but more IL-17. The protective effect of treatment with anti-IL-22 after disease onset is blunted when performing the experiment on *Ifng* ^−/−^ mice. In contrast, mice treated with anti-IL-22 before disease onset exacerbates disease, which is associated with decreased Th1 and unchanged Th17 proportions in draining lymph nodes but increased IL-17 production from cells from the affected joints. In summary, the pathogenic effect of IL-22 during late CIA is dependent on suppression of IFNγ, possibly mediated directly *via* IL-22R expression, which is upregulated on CD4 T cells during this phase of the disease.

A study by another group primarily focused on the influence of IL-22 on autoantibody formation in the context of CIA in C57BL/6 mice but also includes some data on T cells (75). They report that germinal center and autoantibody formation is reduced in *Il22* ^−/−^ mice and IL-22R expression is detected in follicular dendritic cell-like stromal cells. Furthermore, human lymphoid stromal cells produce B cell attracting chemokines CXCL12 and CXCL13 upon stimulation with IL-22. Regarding T cells, they show that, despite having less severe CIA, the *Il22* ^−/−^ mice have increased proportions of Th17 cells in the spleen ten days after immunization. The Th17 cells retain their pathogenic potential, which is shown by assessing secretion of IL-6 in co-cultures with synovial fibroblasts.

#### Psoriasis Arthritis

Psoriasis arthritis is characterized by skin lesions and articular inflammation and is often accompanied by osteoporosis. As with many of the inflammatory diseases Th17 cells have been shown to be critical in psoriasis arthritis (76). Central in the differentiation of Th17 cells is activation of transcription factor STAT3. In a novel model of psoriasis arthritis based on overactive STAT3 function specifically in CD4 T cells, the psoriatic skin phenotype and osteopenia were both ameliorated by either neutralizing IL-17 antibody or genetic deletion of *Il22* (77). The *Il22* ^−/−^ mice had no reduction in total T helper cell infiltration in the skin but had reduced proportions of Th17 cells consistent with a disease promoting role of IL-22. Increased proportions of Tregs but also Th1 cells were seen in the inflamed skin of the *Il22* ^−/−^ mice. Although an improvement in the osteoporosis phenotype was seen in the *Il22* ^−/−^ mice the proportion of IL-17^+^ cells in the bone marrow was increased. Similarly, the proportions of Th1 cells and Tregs were also increased.

#### Encephalomyelitis

Experimental autoimmune encephalomyelitis (EAE) is an animal model of MS and can be induced by immunization with myelin autoantigens, which leads to a T cell driven disease characterized by ascending paralysis. Although *Il22* ^−/−^ mice have no apparent phenotype in EAE experiments (78), mice lacking the endogenous antagonist molecule IL-22BP have less severe disease compared to wild type mice, suggesting a protective role for IL-22 (79). These seemingly contradictory findings can be reconciled if the presence of IL-22BP blocks IL-22 in wild type mice to such a degree that they are indistinguishable from *Il22* ^−/−^ mice. Consistent with the reduced EAE severity, IL-22BP knockout mice have decreased infiltration of Ly6C^+^ inflammatory monocytes in the central nervous system (79). Although not statistically significant, a trend toward less Il-17^+^ T cells infiltrating the CNS in IL-22BP knockout mice is seen. In a follow-up study the role of IL-22BP in EAE was further dissected using an inducible *in vivo* IL-22BP knockdown rat strain. Reducing IL-22BP expression before EAE immunization results in reduced incidence and severity of disease in conjunction with increased proportions of Tregs and decreased proportions of Th1 cells in the lymph nodes that drain the site of immunization (80). Taken together, IL-22 appears to have a protective effect in autoimmune neuroinflammation but, unlike many other animal models discussed in this article, the effect of endogenous IL-22 is normally blocked by IL-22BP. Homeostatic expression of this soluble antagonist molecule is detected in many tissues including secondary lymphoid organs where immune responses are initiated and in the CNS by microglia, making it an interesting pharmacological target in MS and other neuroinflammatory diseases.

#### Dermatitis

In contrast to EAE, IL-22BP knockout mice have more severe disease in an imiquimod induced model of psoriasis (81). This is consistent with the well documented pathogenic role of IL-22 in rodent models of psoriasis (4). The draining lymph nodes of the IL-22BP knockout mice have larger proportions of CD44^+^ CD62L^−^ activated T cells. Also, proportions of both IFNγ producing and IL-17 producing CD8 T cells are elevated compared to wild type mice.

#### Allergic Airway Inflammation

The role of IL-22 has been investigated in a model of atopic dermatitis and allergic asthma in which mice are epicutaneously sensitized to the model antigen ovalbumin followed by intranasal challenge (82). Sensitization alone results in increased serum levels of IL-22 and after intranasal challenge increased *Il22* mRNA as well as increased eosinophil and neutrophil infiltration is detected in the airways. Applying the disease model to *Il22* ^−/−^ mice results in less eosinophils and neutrophils in the airways and reduces airway resistance after methacholine provocation. Cells acquired by bronchoalveolar lavage from *Il22* ^−/−^ mice contain more IFNγ producing type 1 ILCs compared to wild type mice. No change was observed regarding IFNγ^+^ CD4 and CD8 T cells. The authors go on to show that IL-22 synergizes with TNF to drive the neutrophil dominated airway inflammation.

#### Hepatitis

IL-22 has well described protective and regenerative effects on the liver parenchyma after various insults (61, 83–85). In contrast, IL-22 has a disease promoting net effect on chronic hepatitis induced in a mouse strain, transgenically made to express hepatitis B virus antigens, that is treated with anti-CD137 to activate T cells. Using this model, *Il22* ^+/+^ mice have increased disease severity, infiltration of granulocytes and T cells compared to *Il22* ^−/−^ mice (86). Moreover, *Il22* ^+/+^ mice also have an altered balance between T cell responses compared to the *Il22* ^−/−^ mice, with higher hepatic and splenic Th17 numbers but unchanged Th1 numbers. Anti-IL-22 treatment reduces the hepatic stellate cell expression of CXCL10 and CCL20, ligands for CXCR3 and CCR6 respectively, both being chemokine receptors expressed on Th17 cells. IL-22 treated hepatic stellate cells have increased chemotactic potential in a transwell assay and specifically attracts Th17 cells.

#### Atherosclerosis

Th1 and Th17 cells both promote atherosclerosis, whereas Tregs are protective. The role of Th2 cells is unclear. Circulating IL-22 and Th22 cells are both elevated in patients with acute coronary syndrome (87). The role of IL-22 has been investigated using a mouse model in which atherosclerosis is induced by feeding *ApoE* ^−/−^ mice a so called western diet (88). By administering either recombinant IL-22 or neutralizing IL-22 antibody it was shown that IL-22 aggravates atherosclerosis, which was associated with increased macrophage and T cell infiltration in the blood vessels with a proportional increase in Th17 cells. IL-22 receptor is expressed in mouse aortic tissue and its expression is further elevated in the atherosclerosis prone mice.

### Infectious Diseases

#### Chlamydial Lung Infection

Chlamydial organisms are obligate intracellular gram-negative bacteria that can cause pneumonia in humans, which is modeled in mice using *Chlamydia muridarum*. The role of IL-22 has been investigated in this disease model using either administration of recombinant IL-22 or neutralizing IL-22 antibody (89). In both cases IL-22 was shown to improve outcome associated with increased IL-17 production in infiltrating cells in the lungs and spleen *ex vivo* as well as in splenocytes after antigen specific activation *in vitro*. Conflicting results were obtained regarding IFNγ production. Anti-IL-22 treated mice also display lower IL-10 production *ex vivo* and after antigen specific stimulation *in vitro*.

#### Opportunistic Fungal Lung Infection

To mimic an opportunistic fungal lung infection in an immunocompromised patient, mice were treated with a neutrophil-depleting antibody prior to infection with *Aspergillus fumigatus* (90). Furthermore, to investigate the role of gut microbiota in this context, the mice were pre-treated with vancomycin prior to infection, which suppresses intestinal segmented filamentous bacteria and other gram-positive bacteria. Vancomycin pre-treatment did not affect disease severity in that study but did, however, reduce the presence of IL-17 and IL-22 in the lungs. They show that *Il22* ^−/−^ mice have increased colonization of intestinal segmented filamentous bacteria, consistent with Ivanov et al. (13). After infection with *A. fumigatus*, the amount of IL-17 in *Il22* ^−/−^ lung tissue appeared to be increased (*p* = 0.06), an effect that was obliterated by pre-treatment with vancomycin. Vancomycin pre-treatment of wild type mice followed by fecal transplant, neutrophil depletion, and *A. fumigatus* infection results in more Th17 cells infiltrating the lungs after fecal transplants from *Il22* ^−/−^ compared to wild type mice. Transferring serum from wild type mice colonized with segmented filamentous bacteria to uncolonized wild type mice infected with *A. fumigatus* results in a decrease in Th17 cells in the lungs compared to transferring serum from colonized *Il22* ^−/−^ mice. This effect is counteracted by premixing the serum with IL-1Ra (Anakinra). Wild type serum has decreased levels of IL-1α but similar levels of IL-1β compared to *Il22* ^−/−^ serum. Collectively making it plausible that IL-22 modulates serum IL-1Ra ligands leading to accumulation of Th17 cells in the lungs after fungal infection.

#### Tuberculosis

The immune response to *Mycobacterium tuberculosis* varies depending on the infecting strain. The hypervirulent W-Beijing lineage of *M tuberculosis* is a growing health threat that is often associated with human immunodeficiency virus and drug resistance. This infection is modeled in mice with the *M. tuberculosis* strain HN878 (46). In this context, *Il22* ^−/−^ mice have normal susceptibility to infection but have higher bacterial burden in the lungs during the chronic stage. IL-22 deficiency has no impact on alveolar macrophages, monocytes, or recruited macrophages in the lungs during the acute stage of disease but reduces the numbers of IFNγ secreting CD4 and CD8 T cells as well as IL-17 secreting CD8 T cells. However, during the chronic stage the numbers of monocytes and recruited macrophages are lower in the *Il22* ^−/−^ mice consistent with the impaired bacterial clearance. Interestingly, IL-17, which has been shown to be required for protective immunity to this strain (91), is increased in CD8 T cells at this stage despite the poorer outcome. This implies that the lack of IL-22 and associated reduction in IFNγ production and increased bacterial burden are not compensated for by increased IL-17 production.

#### Malaria

Plasma levels of IL-22 are elevated in acute infection with *Plasmodium falciparum* in humans as well as in the murine malaria model *Plasmodium berghei* infection in C57BL/6 mice. *Il22* ^−/−^ mice have earlier onset of cerebral malaria compared to wild type mice despite similar parasite burden in the liver and decreased parasitemia (92). Similar results were seen in experiments using neutralizing IL-22 antibody *in vivo*. Day 3 after infection the *Il22* ^−/−^ mice have increased proportions of IFNγ^+^ cells in the spleen, evident in both CD4 and CD8 T cells as well as γδ T cells. Although no difference in IFNγ expression was observed day 6, IL-17 expression in CD4 T cells as well as γδ T cell is decreased at both time points in *Il22* ^−/−^ mice. APC and CD8 T cell co-cultures using bone marrow derived dendritic cells pulsed with antigen showed that both APCs and CD8 T cells from *Il22* ^−/−^ mice are primed to produce more IFNγ compared to cells from wild type mice. Both CD11c^+^ CD11b^−^ dendritic cells *ex vivo* and bone marrow derived dendritic cells from *Il22* ^−/−^ mice have increased lipopolysaccharide induced expression of CD80 and CD86. Adoptive transfer of splenocytes from OT1 mice, that have ovalbumin specific T cell receptors, into wild type or *Il22* ^−/−^ recipients followed by infection with a transgenic ovalbumin-expressing malaria strain results in more antigen specific T cell proliferation in the *Il22* ^−/−^ recipients.

#### Bacterial Colonization

The potential of IL-22 to influence the crosstalk between the microbiota and the immune system during non-inflammatory conditions in the mouse has been examined. *Staphylococcus epidermidis* was applied to the skin and using both gene-deleted mice and neutralizing antibody, the authors demonstrate IL-22 dependent upregulation of MHC class II expression on keratinocytes, which results in increased numbers of *S. epidermidis* specific Th1 in the skin but no difference was observed regarding Th17 cells (93). Genetic deletion of MHC class II specifically in keratinocytes reduces the Th1 cells but leaves Th17 cells unchanged. The authors speculate that this may be a result of differential requirements of Th1 and Th17 cells to local co-stimulation and chemokines produced by activated keratinocytes.

#### Viral Infection

The role of IL-22 in viral infections is less studied compared to bacterial infections. A study reports that IL-22 is secreted in liver and lymphoid organs within the first few days after intravenous administration of lymphocytic choriomeningitis virus (LCMV) (94). Here IL-22 is expressed mainly by γδ T cells and is dependent on the PI3K/mTOR pathway but not AhR signaling (94). IL-22R was detected on CD45^−^ cells in both the thymus and the spleen. Infecting *Il22* ^−/−^ mice with the LCMV results in increased expression in splenic CD4 T cells of activation marker CD44, chemokine receptor CXCR3, and proliferation marker ki67. Antigenic restimulation of splenic and liver T cells *in vitro* results in higher proportions of IFNγ^+^ CD4 and CD8 T cells. Overexpression of IL-22 leads to the opposite results, confirming that IL-22 dampens IFNγ^+^ T cell responses during acute (7 days) and persistent (60 days) LCMV infection in both lymphoid organs and the liver.

#### Immunization

In a study explicitly designed to study potential indirect effects of IL-22 on T helper cell responses, mucosal immunization with ovalbumin and the adjuvant cholera toxin was performed (95). In *Il22* ^−/−^ mice this results in greater antigen specific T cell responses to mucosal (intrarectal), but not systemic (intraperitoneal) immunization. Polyclonal and antigen specific restimulation *in vitro* of splenic T cells from the mucosally immunized *Il22* ^−/−^ mice results in elevated secretion of IFNγ and IL-17, but no difference was seen after systemic immunization. In this case the proposed mechanism is increased epithelial permeability in the mucosal membrane in the absence of the homeostatic trophic effects of IL-22 allowing more antigen to come in contact with the immune system.

### Other Diseases

#### Multiple Myeloma

Circulating Th17 cells as well as serum levels of IL-22 and IL-17 are elevated in multiple myeloma patients compared to healthy controls, which has spurred investigations into the role of IL-22 in this disease. When peripheral blood mononuclear cells from multiple myeloma patients are cultured under Th1 polarizing conditions in the presence of IL-22 or IL-17, no effect on IFNγ was observed but when the two cytokines are combined IFNγ production is reduced (96). The authors suggest that the elevated circulating IL-22 and IL-17 in multiple myeloma patients may dampen Th1 responses potentially contributing to the observed immune dysfunction in this patient group.

#### Radiation Induced Thymic Injury

IL-22 produced by local lymphoid tissue inducer (LTi) cells after radiation induced thymic injury is essential for the regeneration of thymopoiesis (97). In this disease model, the upregulation of IL-22 in the thymus is dependent on IL-23 from dendritic cells, primarily of the CD103^+^ subset, and may be triggered by the loss of double positive thymocytes. IL-22R subunit *Il22ra1* is expressed on thymic epithelial cells and *in vitro* treatment of these cells with IL-22 results in improved survival and increased proliferation. Administration of IL-22 to irradiated mice with or without subsequent hematopoietic stem cell transplantation results in increased thymic cellularity, including all developing thymocyte subsets and thymic epithelial subsets.

#### Lung Cancer

IL-22 and Th22 cells are elevated in sera and tumor samples from patients with lung cancer and high IL22R1 expression is an indicator of poor prognosis in non-small cell lung cancer. In a study aimed at elucidating the role of IL-22 in tumor-promoting inflammation a *Kras*-induced mouse lung cancer model was used in combination with genetic deletion of *Il22* (98). In the absence of IL-22 lung tumor burden is reduced. Characterization of the bronchoalveolar lavage fluid T cells showed reduced proportions of Tregs and increased proportions of IFNγ^+^ CD4 and CD8 T cells. IL-22 is known to induce STAT3 activation, which has also been observed in non-small cell lung cancer and is furthermore associated with poor prognosis, thus being a plausible mechanism for the effects on tumor burden in the mouse model. However, this would not explain the observed effects of IL-22 on T cell phenotype. The authors propose that pharmacologic targeting of IL-22 may have potential as an add-on therapy to conventional treatments of *KRAS*-mutant lung cancer.

#### Liver Allograft Rejection

Both adaptive and innate immune cells influence acute liver transplant rejection. IFNγ and IL-17 secretion is involved but the role of IL-22 is largely unknown. In a rat model of acute liver allograft rejection treatment with IL-22 neutralizing antibody 12 h before sacrificing the animal day 1, representing ischemia-reperfusion-injury, results in worse liver function (99). In contrast, treatment with IL-22 antibody 24 h before sacrificing the animal on day 7, representing acute rejection, results in improved liver function. At both timepoints IL-22 promoted expression of anti-apoptosis and pro-regeneration associated genes, implying that another mechanism overrides these effects day 7 when clinical outcome is better compared to controls. IL-22 neutralization is associated with increased proportions of Tregs and decreased proportions of Th17 cells in the liver allografts day 7 but no difference was observed day 1. The authors propose that at both timepoints the effect of IL-22 is mediated *via* STAT3. During the ischemia-reperfusion-injury stage the protective effect on hepatocytes by induction of anti-apoptotic and reparative factors lead to better clinical outcome but at the acute rejection stage day 7 induction of hepatocyte chemokine secretion and Th17 type inflammation dominate leading to worse clinical outcome.

#### Graft *Versus* Host Disease

Several studies have examined the role of IL-22 in GvHD. In a model of acute GvHD performed by injecting C57BL/6 splenocytes into F1 progeny of C57BL/6 and D2 mice, neutralizing IL-22 antibody was administered simultaneously and results in increased survival as well as suppressed expansion of donor CD8 T cells and decreased disease-associated depletion of host cells, particularly B cells (100). This is associated with increased proportions of Tregs in the spleen and decreased proportions of IFNγ, IL-4, and TNF secreting CD4 T cells. Splenic CD11b^+^ cells harvested from acute GvHD mice have upregulated IL-22R mRNA as well as protein and cells from IL-22 antibody treated mice have decreased expression of co-stimulatory molecules. Co-cultures with CD11b^+^ cells from IL-22 antibody treated mice with normal C57BL/6 CD4^+^ CD25^−^ T cells promote Treg induction.

The role of IL-22 has also been investigated in a bone marrow transplantation model in which recipient mice were exposed to total body irradiation followed by injection of T cell depleted allogenic bone marrow (101). Treatment with recombinant IL-22 in this model accelerates thymic reconstitution. To induce GvHD the recipient mice were injected with allogenic T cells from the same donor mouse strain. Treatment with recombinant IL-22 has no effect on acute GvHD (day 7) but reduces severity of chronic GvHD (day 60), which is associated with increased numbers of natural Tregs in the thymus and the spleen and decreased numbers of Th1 cells in the spleen. No effect was seen on induced Tregs. The same group has shown using the same acute GvHD model with *Il22* ^−/−^ mice that recipient derived IL-22 reduces severity of disease and improves survival day 30 (102). The *Il22* ^−/−^ mice has higher proportions of Th1 cells in liver, spleen, and intestines. Tregs are reduced but no effect was seen on Th17 cells. IL-22R mRNA was detected in bone marrow derived dendritic cells and stimulation of these with recombinant IL-22 results in reduced expression of CD80 and IFNγ. Compared to wild type bone marrow derived dendritic cells *Il22* ^−/−^ counterparts co-cultured with wild type T cells results in higher proportions of Th1 cells and lower proportions of Tregs.

## Concluding Remarks

**Figure 2:**
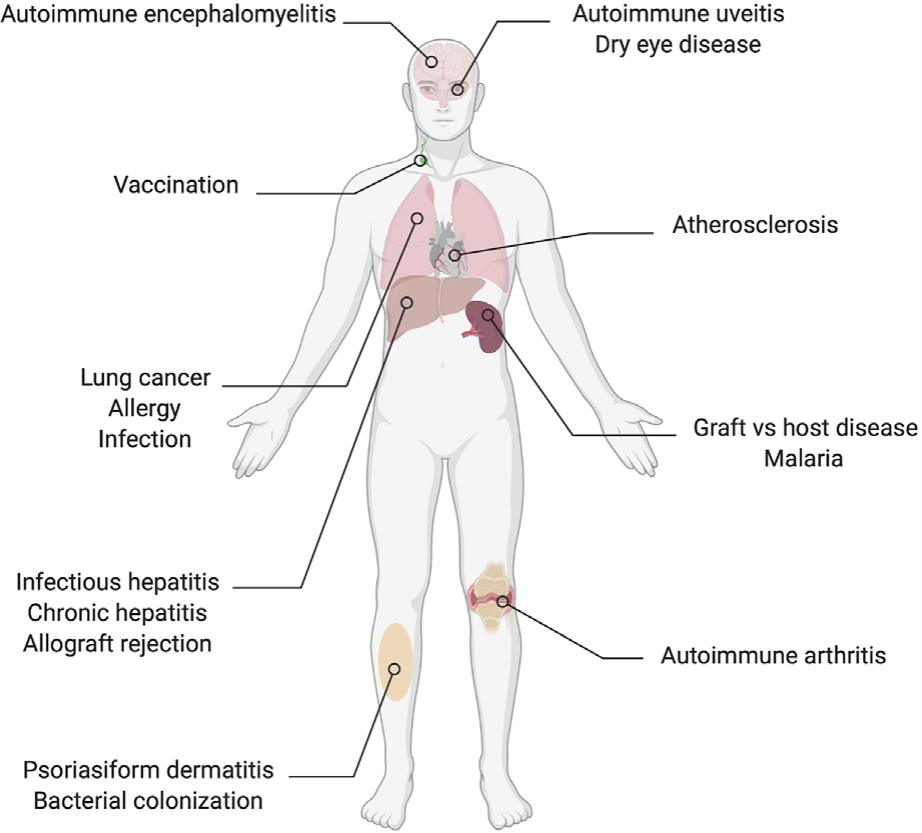
Organs and conditions in which there are reported effects of IL-22 on the Th1/Th17 axis.

**Figure 3:**
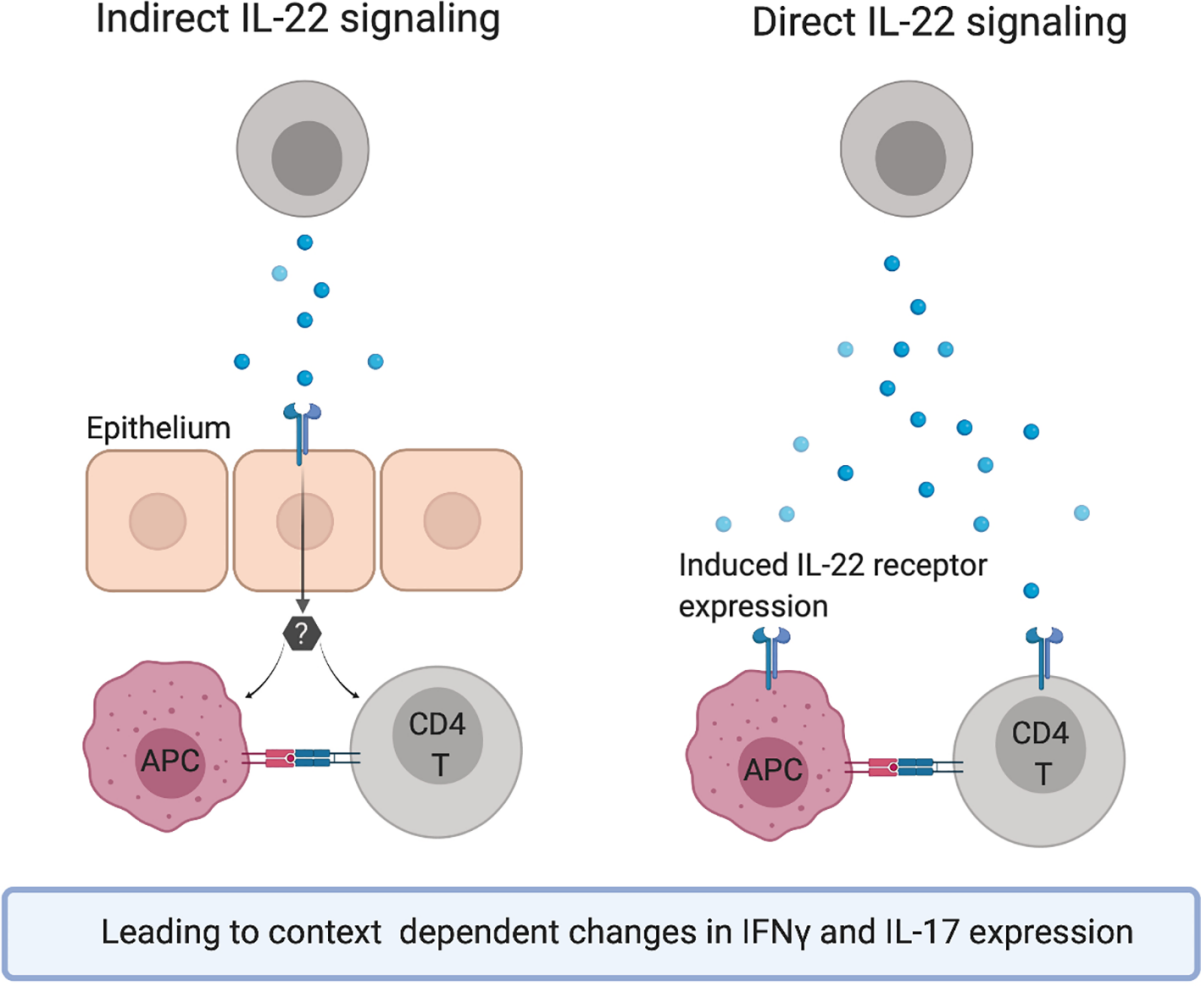
Hypothetical mechanisms of action for the observed effects of IL-22 on the Th1/Th17 axis.

Published data that demonstrate direct or indirect effects of IL-22 on T cell polarization or secretion of signature cytokines in the context of a wide range of diseases are compiled in this review (**Table 2**, **Figures 2** and **3**). The observed effects are not uniform, but the data suggest that IL-22 signaling often results in reduced Th1 type responses and it may also contribute to resolution of inflammation by promoting Tregs or IL-10 secretion.

**Table 2:** The influence of IL-22 on the Th1/Th17 axis.

Phenotype	Context	Effect	Influence of IL-22	References
Less IFNγ	Healthy human	–	Decreases IFNγ during Th1 differentiation of PBMCs when combined with IL-17	Prabhala et al. (96)
	EAU	pos	Decreases splenic T cell IFNγ *in vitro*	Ke et al. (68)
	Murine malaria	pos	Decreases splenic T cell IFNγ *ex vivo* and *in vitro*	Sellau et al. (92)
	CIA (treatment after onset)	neg	Decreases IFNγ or Th1 cells in spleen, draining lymph nodes, and APC/T cell co-culture	Justa et al. (64)
	CIA	pos	Decreases IFNγ after polyclonal activation of splenocytes *in vitro*	Sarkar et al. (74)
	EAE	pos	Decreases Th1 cells in draining lymph node cells 7 days after immunization	Lindahl et al. (80)
	GvHD (bone marrow)	pos	Decreases Th1 cells in the spleen day 60	Pan et al. (101)
	GvHD (splenocytes)	pos	Decreases donor Th1 cells in target tissues	Pan et al. (102)
	Allergic airway inflammation	pos	Decreases IFNγ in BAL cells	Leyva-Castillo et al. (82)
	Lung cancer	neg	Decreases Th1 and IFNγ^+^ cytotoxic T cells in lung tumor tissue	Khosravi et al. (98)
	Vaccination	pos	Decreases IFNγ in restimulated splenocytes	Budda and Zenewicz (95)
	Psoriasis arthritis model	neg	Decreases Th1 cells in the skin and bone marrow	Yang et al. (77)
	LCMV infection	neg	Decreases Th1 and IFNγ^+^ cytotoxic T cells in the liver and spleen	Yi et al. (94)
More IFNγ	Mycobacterial lung infection	pos	Increases IFNγ^+^ T cells in the lungs during the acute stage of disease only	Treerat et al. (46)
	CIA (treatment before onset)	pos	Increases Th1 cells in draining lymph nodes	Justa et al. (64)
	Psoriasiform dermatitis	neg	Increases IFNγ^+^ cytotoxic T cells in the draining lymph nodes after polyclonal stimulation *in vitro*	Lindahl et al. (81)
	Staphylococcal colonization on skin	–	Increases antigen specific Th1 cells	Tamoutounour et al. (93)
	GvHD	neg	Increases Th1 cells in the spleen	Wu et al. (100)
Less IL-17	Untreated mice	–	Decreases Th17 cells in the intestines	Qiu et al. (73)
	EAU	pos	Decreases splenocyte IL-17 expression *in vitro*	Ke et al. (68)
	Pulmonary fungal infection	–	Decreases Th17 cells in the lungs	McAleer et al. (90)
	Murine malaria	pos	Decreases Th17 cells in the spleen	Sellau et al. (92)
	CIA	pos	Decreases splenocyte IL-17 expression *ex vivo*	Corneth et al. (75)
	CIA (treatment before onset)	pos	Decreases Th17 cells in joints	Justa et al. (64)
	Vaccination	pos	Decreases IL-17 production in antigen stimulated splenocytes *in vitro*	Budda and Zenewicz (95)
	Psoriasis arthritis model	neg	Decreases Th17 cells in the bone marrow	Yang et al. (77)
	Dry eye disease model	pos	Decreases Th17 cells in the eyes	Ji et al. (35)
More IL-17	Mycobacterial lung infection	pos	Increases IL-17^+^ cytotoxic T cells during the acute and chronic phase of the disease	Treerat et al. (46)
	Chlamydial lung infection	pos	Increases Th17 cells the lungs and spleen	Peng et al. (89)
	Chronic hepatitis	neg	Increases Th17 cells in the liver and spleen	Zhao et al. (86)
	CIA (treatment after onset)	neg	Increases Th17 cells in the joints and splenic APC/T cell co-cultures	Justa et al. (64)
	CIA	pos	Increases Th17 cells in the spleen after antigen specific restimulation	Sarkar et al. (74)
	Psoriasiform dermatitis	neg	Increases IL-17^+^ cytotoxic T cells in the draining lymph nodes after polyclonal stimulation *in vitro*	Lindahl et al. (81)
	Psoriasis arthritis model	neg	Increases Th17 cells in the skin	Yang et al. (77)
	Atherosclerosis	neg	Increases Th17 cells in the blood vessel	Lin et al. (87)
	Liver allograft rejection – acute rejection	neg	Increases Th17 cells in the allografted liver	Zhang et al. (99)
More IL-10	EAU	pos	Increases splenic T cell IL-10 expression *in vitro*	Ke et al. (68)
	CIA	pos	Increases splenic T cell IL-10 expression *in vitro*	Sarkar et al. (74)
	Chlamydial lung infection	pos	Increases T cell IL-10 expression in the lungs, spleen, and lymph nodes	Peng et al. (89)
Less Tregs	GvHD	neg	Decreases Tregs in the spleen	Wu et al. (100)
	Psoriasis arthritis model	pos	Decreases Tregs in the skin and bone marrow	Yang et al. (77)
	Liver allograft rejection – acute rejection	neg	Decreases Tregs in the allografted liver tissue	Zhang et al. (99)
More Tregs	EAE	pos	Increases Tregs in the draining lymph nodes	Lindahl et al. (80)
	GvHD (bone marrow)	pos	Increases Tregs in the spleen and thymus.	Pan et al. (101)
	GvHD (splenocytes)	pos	Increases Tregs in the spleen, liver, and small intestines	Pan et al. (102)
	Lung cancer	neg	Increases Tregs in lung tumor tissue	Khosravi et al. (98)

T cell polarization or cytokine expression in experiments in which IL-22 signaling was specifically manipulated. The effect for each disease model is assigned as pos if IL-22 results in less severe disease and neg if IL-22 results in more severe disease. PBMC, peripheral blood mononuclear cells; EAU, experimental autoimmune uveitis; CIA, collagen induced arthritis; APC, antigen presenting cell; EAE, experimental autoimmune encephalomyelitis; GvHD, graft versus host disease; BAL, bronchioalveolar lavage; ILC, innate lymphoid cell; LCMV, lymphocytic choriomeningitis virus; Tregs, regulatory T cells.

IL-22 has an important role in the response to bacterial pathogens in several models of infectious diseases including Ci*trobacter rodentium* colitis (30, 40) and *Klebsiella pneumonia* (103) as well as *Mycobacterium tuberculosis* infections of the lungs (46). The proposed mechanisms have primarily been maintenance of physiological barriers and induction of antimicrobial peptides. Considering this, it is somewhat surprising that available data frequently describe an inhibitory effect of IL-22 on Th1 immune responses. In contrast, if one considers the inflammatory diseases, an inhibitory effect of IL-22 on inflammatory T cell responses is consistent with the observed net effect of IL-22 on most models. The notable exception is psoriasis and other dermatitides where IL-22 has a pathogenic effect, which likely involves direct actions of IL-22 on keratinocytes leading to excessive proliferation, aberrant maturation as well as induction of inflammatory mediators (6, 11, 104–106).

Although not covered in this review, several studies have shown that IL-22 can promote B cell responses. CXCL13 is a central chemokine in B cell immune responses and is expressed in follicles of lymphoid tissues where it attracts B cells *via* the receptor CXCR5. *In vivo* neutralization of CXCL13 in mice reduces B cell recruitment to lymphoid follicles and inhibits formation of germinal centers (107). CXCL13 can be induced by IL-22 in tertiary lymphoid follicles (108) offering a plausible explanation to the elevated antibody levels in response to IL-22. In mice, IFNγ promotes isotype switching to IgG2a or IgG2c (109). The IL-22 mediated decrease in Th1 responses would thus be expected to be associated with a reduction in these isotypes which was the case in the study of Geboes et al. (66), but not in Justa et al. (64) or Corneth et al. (75). Although Th2 cells have not frequently been assessed in the included studies one can speculate that IL-22 induces a shift in the Th1/Th2 balance toward a Th2 and humoral immune response.

Both the proliferative and anti-apoptotic effects on epithelia and other tissues commonly attributed to IL-22 signaling as well as a potential inhibitory effect on T cell responses may lead to a permissive environment for tumor growth. IL-22 has been reported to both increase and decrease formation of tumors depending on tissue and model system but a tumor-promoting effect is more commonly observed (72). Interestingly, constitutive deletion of the IL-22 antagonist molecule IL-22BP in mice does not result in more tumor formation in steady state conditions but in the context of chronic colitis the unrestrained IL-22 signaling results in increased incidence of colon tumors. On the other hand, another study demonstrated a protective role of IL-22 in inflammation-induced colon tumors by improving the cellular response to DNA damage (110).

In summary, IL-22 signaling often reduces Th1 type immune responses. This characteristic of IL-22 may act in synergy with its protective effects on IL-22R expressing tissue cells to reduce collateral damage in the context of infection and inflammation. The long-term risk of tumor growth needs to be counterbalanced, which is the likely role of the endogenous IL-22 antagonist IL-22BP that is constitutively expressed in many tissues.

## Author Contributions

HL reviewed the literature and drafted the manuscript. TO revised the manuscript and approved the final version. All authors contributed to the article and approved the submitted version.

## Conflict of Interest

The authors declare that the research was conducted in the absence of any commercial or financial relationships that could be construed as a potential conflict of interest.

## Abbreviations

IL, interleukin; RA, rheumatoid arthritis; MS, multiple sclerosis; IL-22R, IL-22 receptor; NKT cell, natural killer T cell; ILC, innate lymphoid cell; γδ T cell, gamma delta T cell; DC, dendritic cell; AhR, aryl hydrocarbon receptor; RORγt, RAR-related orphan receptor gamma t; TLR, Toll-like receptor; TGF-β, transforming growth factor beta; ICOS, inducible T cell co-stimulator; IL-22BP, IL-22 binding protein; TNF, tumor necrosis factor; IFNγ, interferon gamma; MAIT cell, mucosa associated invariant T cell; LTi cell, lymphoid tissue inducer cell; EAU, experimental autoimmune uveitis; APC, antigen presenting cell; CIA, collagen induced arthritis; LCMV, lymphocytic choriomeningitis virus; GvHD, graft *versus* host disease.
